# Secretoneurin Gene Therapy Improves Blood Flow in an Ischemia Model in Type 1 Diabetic Mice by Enhancing Therapeutic Neovascularization

**DOI:** 10.1371/journal.pone.0074029

**Published:** 2013-09-23

**Authors:** Wilfried Schgoer, Markus Theurl, Karin Albrecht-Schgoer, Verena Jonach, Bernhard Koller, Daniela Lener, Wolfgang M. Franz, Rudolf Kirchmair

**Affiliations:** Internal Medicine III (Cardiology and Angiology), Medical University Innsbruck, Innsbruck, Austria; University of Bristol, United Kingdom

## Abstract

Deficient angiogenesis after ischemia may contribute to worse outcome of peripheral arterial disease in patients with diabetes mellitus. Based on our previous work where we demonstrated that Secretoneurin (SN) is up-regulated under hypoxic conditions and enhances angiogenesis, we analyzed the therapeutic potential of SN gene therapy using a model of severe hind limb ischemia in streptozotocin-induced diabetic mice (STZ-DM). After induction of hind limb ischemia, blood flow was assessed by means of laser Doppler perfusion imaging (LDPI) and increased blood perfusion in the SN-treated animal group was observed. These results were complemented by the clinical observation of reduced necrosis and by an increased number of capillaries and arterioles in the SN-treated animal group. *In vitro*, we found that SN is capable of promoting proliferation and chemotaxis and reduces apoptosis in HUVECs cultured under hyperglycemic conditions. Additionally, SN activated ERK, eNOS and especially AKT as well as EGF-receptor in hyperglycemic HUVECs. In conclusion, we show that SN gene therapy improves post-ischemic neovascularization in diabetic mice through stimulation of angiogenesis and arteriogenesis indicating a possible therapeutic role of this factor in ischemia-related diseases in diabetic patients.

## Introduction

Diabetes mellitus (DM) is characterized by microvascular disease leading to retinopathy, nephropathy and neuropathy but also by macrovascular atherosclerotic complications like coronary heart disease (CHD), peripheral arterial disease (PAD) and involvement of cerebral arteries resulting in increased morbidity and mortality [1]. Diabetic patients present a different pattern of PAD and CHD compared to non-diabetics, characterized by involvement of small, peripheral arteries and by reduced capacity to promote collateral blood vessel growth. In PAD these defects result in severe manifestations of vascular disease called critical limb ischemia (CLI) leading to ischemic ulcers and/or gangrene with potential limb loss [2]. Although revascularization with peripheral bypass grafting and percutaneous approaches is the current standard of care for CLI, it has been shown that revascularization often is not successful and diabetic patients may have even worse outcome following vascular interventions. Additionally, a substantial number of patients are unsuitable for vascular interventions, thus, there is a need for alternative treatment strategies in diabetic patients with CLI [3]–[6]. Numerous mediators, including growth factors and transcription factors have been reported to augment chronic ischemia-induced angiogenesis in animal models, a procedure called therapeutic angiogenesis [7]. Although growth-factor based pro-angiogenic clinical trials have shown relatively limited outcome [6]–[12], therapeutic neovascularization remains an attractive treatment modality for these patients.

Neovascularization is believed to occur via 3 possible mechanisms: the sprouting of preexisting resident endothelial cells (angiogenesis), maturation and enlargement of the preexisting small vessels through vascular remodeling (arteriogenesis), and the recruitment of bone marrow-derived endothelial progenitor cells (vasculogenesis). There is increasing evidence that all of the processes of neovascularization are impaired in diabetic ischemia [13], [14].

We demonstrated that the neuropeptide secretoneurin (SN) induces angiogenesis and postnatal vasculogenesis in a cornea neovascularization assay [15], [16]. *In vitro*, SN induced capillary tube formation and exerted proliferative, chemotactic and antiapoptotic effects on endothelial cells (EC). Additionally we observed that SN is up-regulated in skeletal muscle cells by hypoxia in-vitro and in-vivo [17]. Furthermore, we generated a SN plasmid therapy vector and showed that gene therapy with SN induces therapeutic angiogenesis, arteriogenesis, and vasculogenesis in the mouse hind limb ischemia model [18]. Based on therapy with this plasmid vector, we could recently also provide evidence for improvement of heart function in a rat model of myocardial infarction [19].

This study aims to elucidate the proliferative and antiapoptotic effects of SN on endothelial cells under hyperglycemic conditions. Additionally, our investigations sought to determine the effectiveness of SN gene therapy on neovascularization in a diabetic murine model of hind limb ischemia leading to a potentially novel therapeutic strategy for vascular disease in diabetes.

## Methods

### SN peptide and gene therapy vector

SN peptide was purchased from Neosystems. Expression vector pAAV-MCS was purchased from Stratagene. Plasmids were constructed as published previously [18].

### Induction of diabetes in mice

All protocols described in this study were approved by the Austrian Committee for Laboratory Animal Use and Care. All measurements and analyses were performed by investigators blinded to the treatment of the animals.

Female 8–12-week-old C57BL/6N mice were used for experiments. All animals were allowed free access to the same standardized diet and water during the entire study. Type 1 diabetes was induced by intraperitoneal injection of 50 mg/kg streptozotocin (STZ, Sigma), diluted in citrate buffer (0.05 mol/L, pH 4.5), for 5 consecutive days. STZ generates significant, predictable, and reproducible hypoinsulinemia and profound hyperglycemia within 1 week after treatment. One week after the fifth injection, non-fasting glucose levels were monitored in blood obtained from the tail vein, by an Accu-Check glucometer (Roche). Mice with hyperglycemia (>300 mg/dL) were considered diabetic and included in the study and surgery was performed 3 weeks later. An elevated glucose level was found in 90% of mice injected with STZ.

### Hind limb ischemia model and perfusion measurement

Prior to surgery, mice were anaesthetised by intraperitoneal injection of 80 mg/kg ketamine and 16 mg/kg xylazine. Briefly, the left femoral artery was exposed, ligated with 5-0 silk ligatures, and excised. For *in-vivo* gene transfer, mice were injected with plasmids expressing SN (p-SN, 50 µg) or plasmids containing the empty vector (p-CTR, 50 µg) into thigh and calf muscles immediately after surgery. Plasmids were dissolved in a volume of 100 µl and injections were performed at 2 sites of the thigh and 1 site at the calf muscle. Limbs were scanned with a laser Doppler perfusion imager (LDPI, Moor Instruments) preoperatively and postoperatively on days 0, 7, 14 and 28 after surgery. For perfusion studies, mice were anaesthetised and fur was removed using depilatory cream. Mice were kept on a heated surface (37°C) to minimize the effects of ambient temperature on measurements. The mean perfusion of each limb was determined, and the ratio between the ischemic and the control limb was calculated to gain relative perfusion. Results are expressed as mean perfusion ± SEM. Peri-operative mice received piritramide (10 mg/kg body weight) and postoperative tramadolhydrochloride (2.5 mg/100 ml drinking water) for the first seven days after operation. The physical condition of the animals was evaluated two times per day.

Assessment of ischemic tissue damage and loss was performed as described previously with small alterations (1 = limb salvage, 2 = skin necrosis, 3 = amputation) [20]. Outcome of all mice were observed and recorded on 28 days after induction of hind limb ischemia.

### Measurement of capillary and arteriole density in the ischemic limb

For tissue staining, mice were sacrificed and ischemic limb tissues were retrieved after 4 weeks. Fresh tissue was embedded in OCT compound (TISSUE-TEK®, Sakura Finetek) and snap-frozen in liquid nitrogen. Tissues were sliced into 5-µm sections. Vascular endothelial cells were identified by staining with CD31 (Pharmingen) and for assessment of arteriole density sections were stained with a mouse monoclonal anti-α-smooth muscle actin (anti-α-SMA) antibody (Pharmingen) as described [15]. For fluorescent microscopy appropriate secondary antibodies (Alexa 488 for α-SMA and Alexa 594 for CD31; both from Invitrogen 1∶200) were used. Adductor muscle samples of each leg were divided into 2 parts and capillaries (CD31 positive) and arterioles (α-SMA-positive cells surrounding the whole circumference of the vessel) were counted in five sections of each part and are expressed as capillary and arteriole density per high power field (HPF).

### Aortic ring assay

For the aortic ring assay we used a slightly modified protocol published by Baker et al. [21]. Mice were killed by cervical dislocation and the mouse-surface was sterilized using 70% ethanol. Consequently the ventral skin and afterwards the thoracic cavity were opened in a laminar flow. Sterile forceps and scissors were used for the experiment. Afterwards heart and lungs were removed, the aorta exposed and gently removed from the spinal column. Consequently the aortas were placed in petri-dishes containing ice cold PBS. Using a dissecting microscope fatty tissue and side-branches were removed, aortas flushed and cut into rings of 1 mm. Afterwards aortic rings were embedded in pH-adjusted bovine collagen solution type-1 (Purecol) and incubated in a humified atmosphere (37°C, 5% CO_2_) for about one hour. Thereafter, the embedded rings were incubated with dilution medium (EBM2 from Lonza +1.5% fetal bovine serum FBS, Biochrom AG) or SN 10 ng/ml and VEGF 50 ng/ml with 25 mM D-glucose or mannitol as negative control. The medium was changed on day 3 and then every other day until the end of the experiment. 24 well plates were used for the experiments. Cells migrated in the area around the aortic ring were counted on displayed days. Isolectin B4 (Vectorlabs) staining was used to identify cells as endothelial cells. Three independent experiments with quadruplicates for each condition were performed.

### Cell culture experiments

For our experiments human umbilical vein endothelial cells (HUVEC) between passage 2 and 5 were used. Cells were purchased from PromoCell and cultured in EBM-2 (Lonza). To simulate hyperglycemia in-vitro, cells were incubated with 25 mM D-glucose, 24 hours prior to experiments. To exclude the influence of osmotic pressure on the behavior of HUVECs, 25 mM mannitol was established as negative control. Consequently, cells were used for experiments.

### Proliferation Assay

The influence of SN on HUVEC proliferation under hyperglycemic conditions was determined by BrdU incorporation (Cell Proliferation ELISA BrdU Kit, Roche). Briefly, cells were incubated with dilution medium (medium without supplements containing 1.5% FBS, supplemented with 25 mM mannitol or glucose), VEGF 50 ng/ml, SN 10 ng/ml or SN 100 ng/ml for 16 hours. Afterwards cells were labeled with BrdU for 6 hours and processed as suggested by the manufacturer. Signals were analyzed using a luminometer (Anthos, Lucy1). For each condition 6 wells were used. Three independent experiments were performed.

### Apoptosis Assay

For apoptosis assays HUVEC were incubated with medium without supplements containing 25 mM mannitol or glucose with VEGF 50 ng/ml, SN 10 ng/ml or SN 100 ng/ml for 24 hours. TUNEL (Terminal deoxynucleotidyl transferase dUTP nick end labeling) assays were performed according to the manufacturer's instructions (Roche) and cells positive for TUNEL staining and for DAPI staining were counted. Results are expressed as % of TUNEL positive cells of all DAPI (total cell number) stained cells. For each condition quadruplicates were used. Three independent experiments were performed.

### Chemotaxis Assay

The migratory response of HUVEC was measured using a modified 48-well Boyden chemotaxis chamber in which an 8 µm pore sized cellulose nitrate filter (Sartorius, Göttingen, Germany) separated the upper and the lower chamber. Cells were detached from the tissue flask with 0.05% trypsin and EDTA (Gibco) and re-suspended at a density of 3×10^5^ cells/well in chemotaxis medium (medium without supplements containing 0.75% FBS and 25 mM mannitol or glucose) before being placed in the upper wells of the chemotaxis chamber (Neuroprobe, Bethesda, Maryland). Migration into the filter was quantified by measuring microscopically the distance from the surface of the filter to the leading front of the cells. Data are expressed as relative chemotaxis index, which is the ratio between the distance of migration towards test attractants and that toward control medium into the filters. For each condition quadruplicates were used. Three independent experiments were performed.

### Western blotting and RTK profiler assay

HUVEC were plated on 60 mm tissue culture dishes and starved for 12 hours in serum free medium (supplemented with 25 mM mannitol or glucose). Thereafter, cells were stimulated with SN 10 ng/ml for different time periods and processed for western blotting. Antibodies against phospho p-44/42 MAPK (Cell signaling), phosho AKT (Cell signaling), phospho eNOS (BD) and α-tubulin (Cell signaling) were used. Corresponding horse radish peroxidase conjugated secondary antibodies were purchased from Jackson. Western blots were detected by chemiluminescence using ECL solutions from Pierce (Thermo Scientific).

Activation of several receptor tyrosine kinases (RTK) were analyzed using human phospho-RTK array kits (R&D, ARY001B) as suggested by the manufacturer.

### Statistical analysis

All results are expressed as mean ± SEM. Statistical comparisons between groups were performed by Student-*t* test. Probability values <0.05 were considered statistically significant. All experiments were repeated at least in triplicate.

#… p<0.05; +… p<0.01; §… p<0.005; *… p<0.001;

## Results

### Physiological data

**Figure 1:**
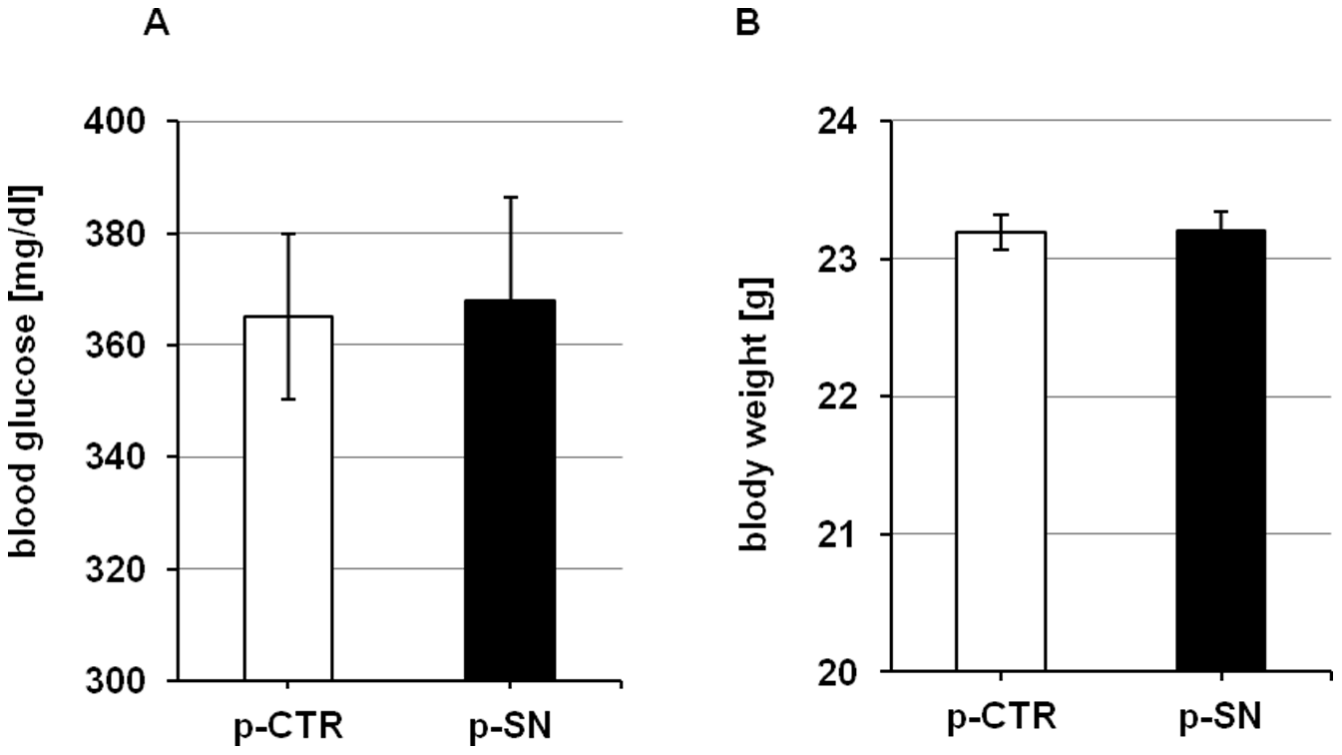
Effects of SN on metabolic parameters. No significant changes in (A) blood glucose level and (B) body weight were found between p-SN and p-CTR treated animals after 28 days.

DM was induced in C57BL/6 mice by intraperitoneal injection of STZ for 5 consecutive days. Fasting blood glucose concentration was measured in all mice before hind limb ischemia operation to confirm a diabetic phenotype and was 359.1±15.7 mg/dL (n = 24). Hind limb ischemia and treatment with p-SN or p-CTR did not affect blood glucose levels (p-CTR vs. p-SN on day 28: 365.4±14.8 mg/dL vs. 367.9±18.3 mg/dL) as shown in figure 1A. Weight did not differ between groups (p-CTR vs. p-SN on day 28: 23.2±0.1 g vs. 23.2±0.1 g, figure 1B).

### Effects of SN gene therapy in the mouse hind limb ischemia model

**Figure 2:**
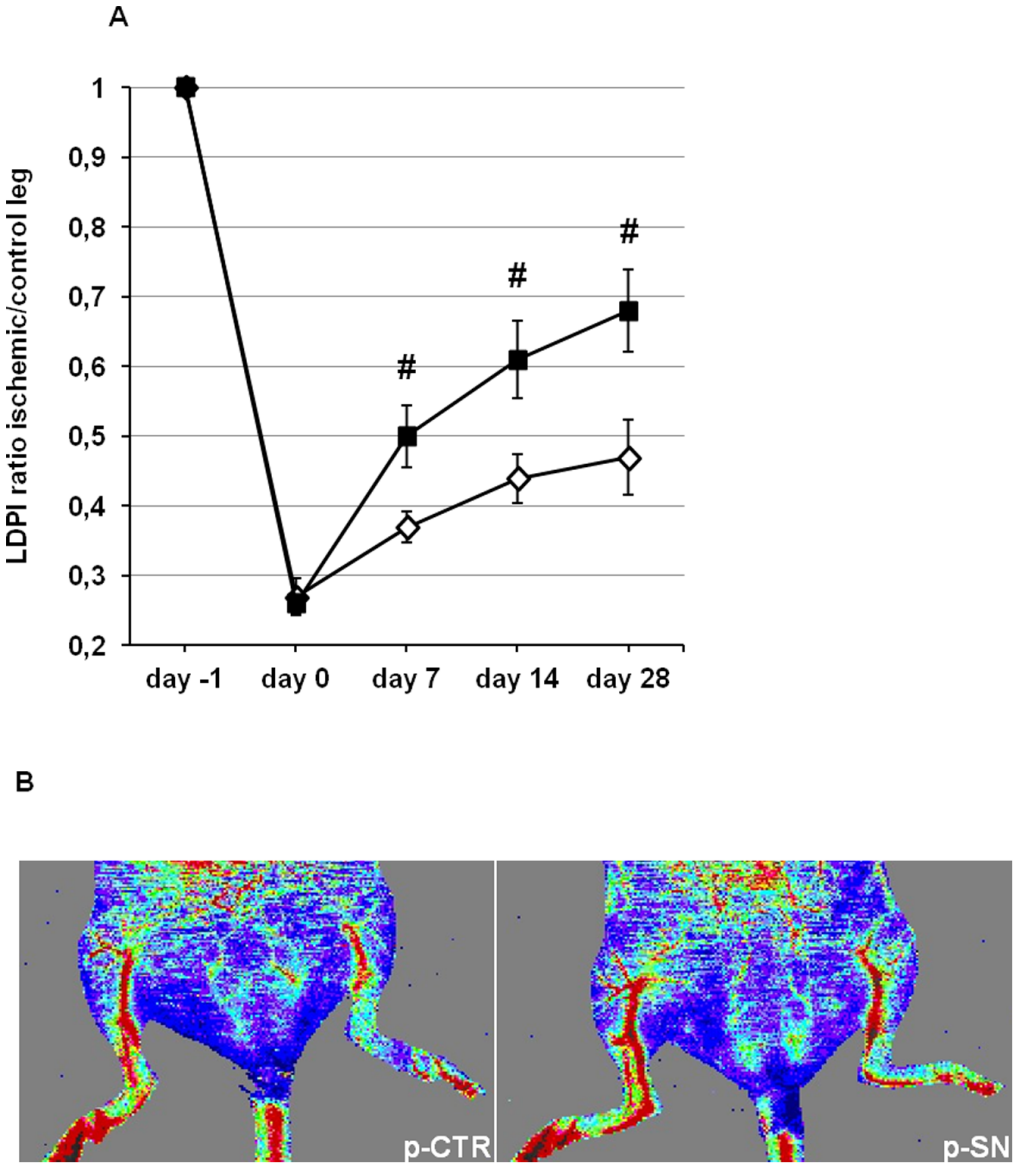
Secretoneurin gene therapy improves blood flow monitored *in vivo* by LDPI in diabetic mice. (A) Time course of hind limb blood flow measured by LDPI is expressed as ischemic-to-control leg ratio. Treatment with p-SN significantly improved perfusion 7, 14 and 28 days after induction of ischemia compared to p-CTR: #, p<0.05. (B) Representative pictures of the ischemic (left) and non ischemic (right) hind limbs on day 28 after ischemia induction. In color-coded images treatment with p-SN improves perfusion where red indicates normal perfusion and blue indicates a marked reduction in blood flow in the ischemic hind limb.

To evaluate the effect of SN gene therapy on blood perfusion in diabetic mice, LDPI was performed before, immediately after and on days 7, 14 and 28 after hind limb ischemia (figure 2A). Pre-operatively, the ratio of blood flow between the two hind limbs in all animals used in this study was 1.0. Immediately after induction of hind limb ischemia, the ratio of the blood flow between the ischemic and normal limb was 0.27±0.03 in the p-CTR and 0.26±0.02 in the p-SN treated group. In mice injected with control-plasmid, blood flow recovered after 28 days to a ratio ischemic/non ischemic limb of 0.47±0.05, whereas in SN-plasmid injected mice LDPI ratio after 4 weeks was 0.68±0.06 (p<0.05 vs. p-CTR; n = 10). Also, after 1 and 2 weeks, SN-treated animals exerted a significantly higher blood perfusion (p<0.05 for both time points vs. p-CTR; n = 10). Thus, blood flow recovery was severely impaired in these diabetic animals and could be markedly improved by SN treatment. Representative LDPI pictures after 28 days are shown in figure 2B.

**Figure 3:**
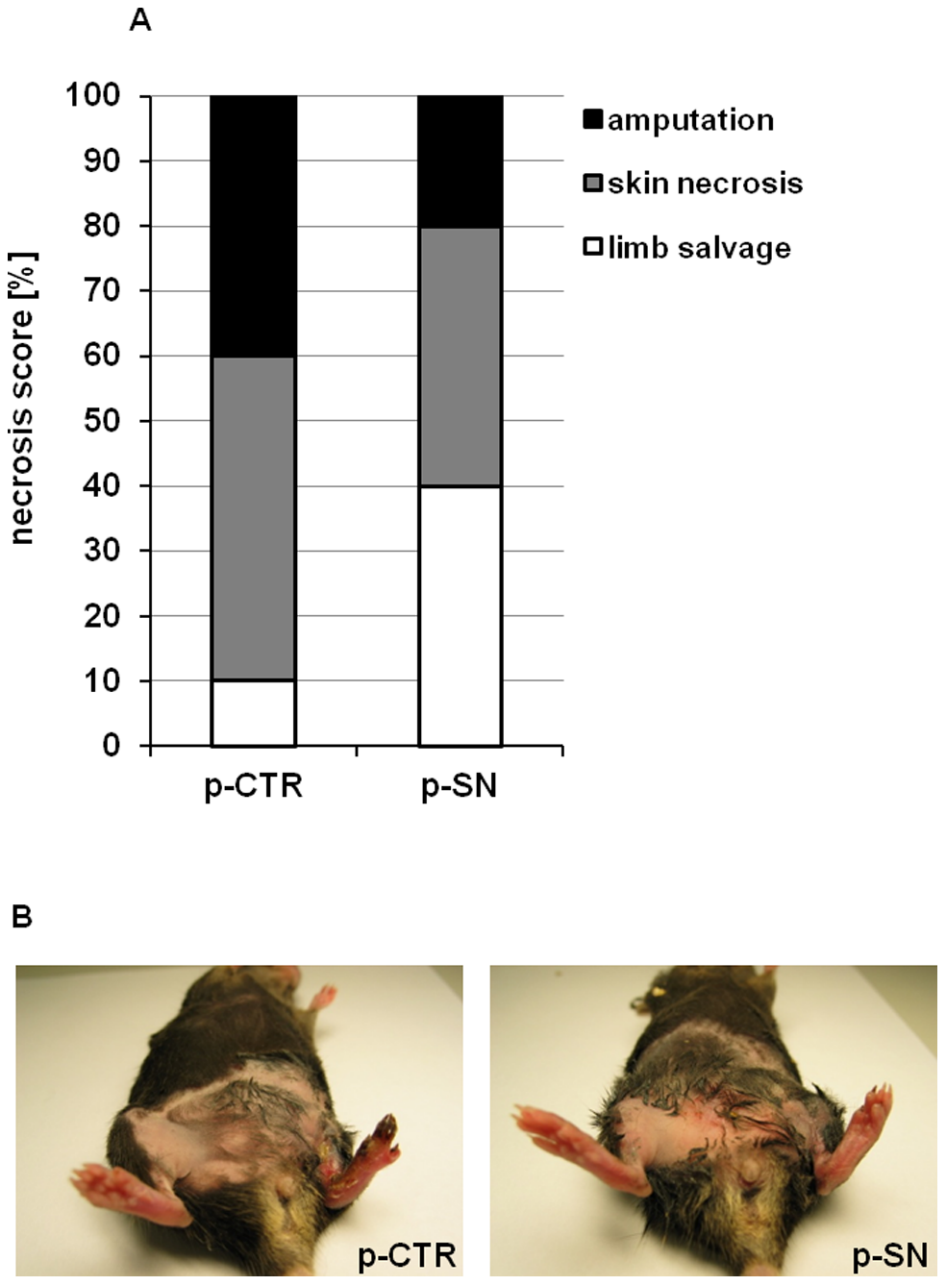
Secretoneurin gene therapy rescues ischemic tissue defects in a diabetic PAD model. (A) Necrosis score at day 28 is shown as % mice with saved limbs (white), % mice with skin necrosis (grey) and % mice with amputations (black). Treatment with p-SN significantly inhibited ischemic tissue loss (necrosis and amputation). p<0.05. (B) Representative pictures of the ischemic (left) and non ischemic (right) limbs on day 28 after ischemia induction and treatment with p-SN or p-CTR.

In terms of clinical outcome, necrosis of ischemic limbs was evaluated. At 4 weeks, 90% of p-CTR treated animals showed signs of tissue defects with skin necrosis (50%) or amputation (40%), whereas only in 60% of SN-treated mice tissue defects were observed (skin necrosis: 40% and amputation: 20%; p<0.05 for tissue defects SN treatment vs. p-CTR using Fischer's exact test; n = 10; figure 3A). SN gene transfer prevented limb necrosis in STZ-DM mice as illustrated in figure 3B.

### SN gene therapy promotes angiogenesis and arteriogenesis

**Figure 4:**
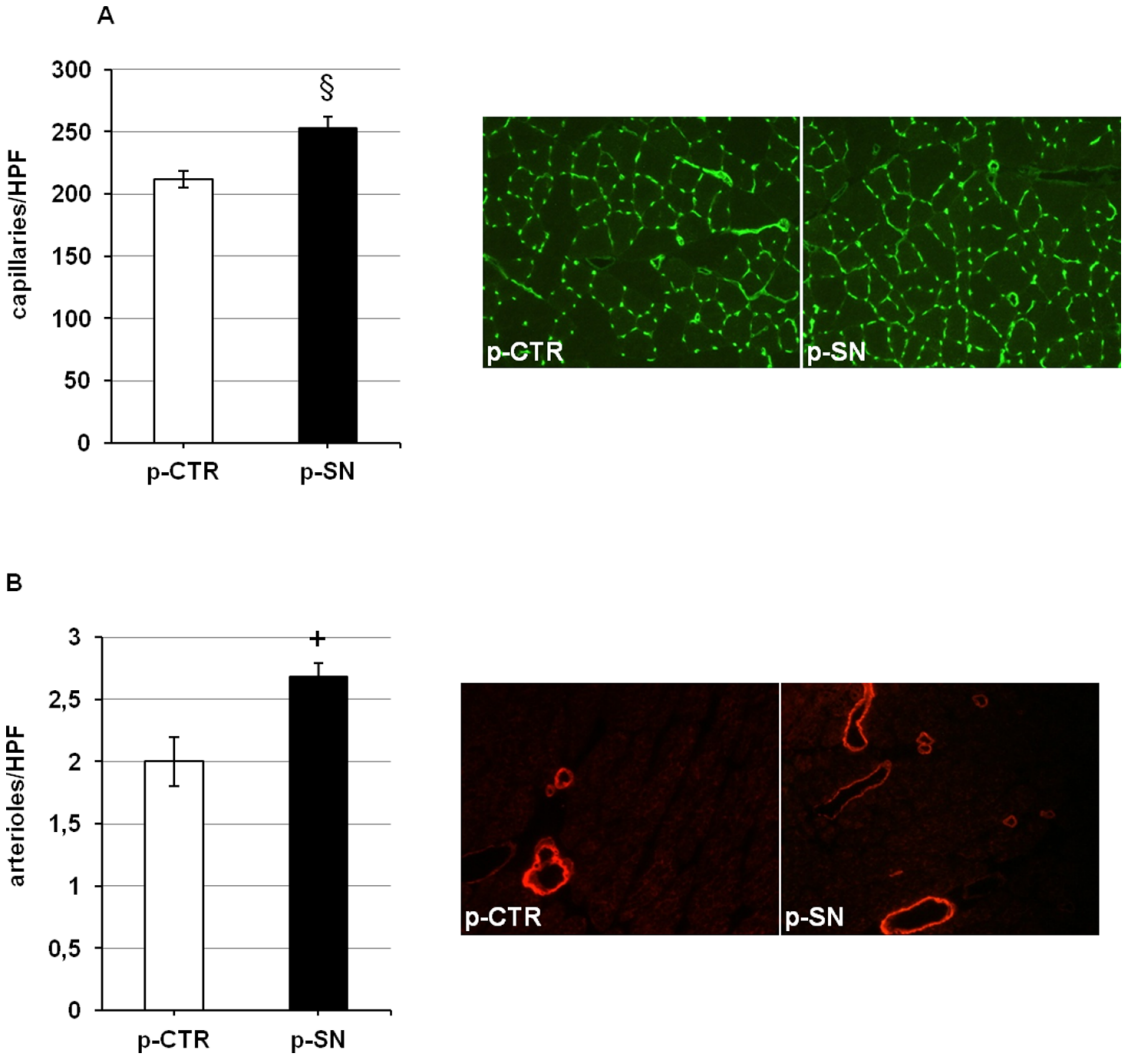
Secretoneurin gene therapy increases number of capillaries and arterioles in ischemic hind limbs. (A) Quantification of capillary number per high power field (HPF) in ischemic muscle tissue treated with p-CTR or p-SN, 28 days after induction of ischemia, and corresponding representative images of staining with CD31 for identification of capillaries. Treatment with p-SN significantly increases number of capillaries. §, p<0.005. (B) Quantification of arteriole number per high power field (HPF) in ischemic muscle tissue treated with p-CTR or p-SN, 28 days after induction of ischemia, and corresponding representative images of staining for alpha- smooth muscle acting. Treatment with p-SN significantly increases number of arterioles/arteries. +, p<0.01.

To determine the effect of SN gene therapy on *in vivo* angiogenesis, we performed staining of capillaries with CD31. Quantification and representative photographs of immunohistochemical sections are shown in figure 4A. Administration of p-SN increased capillary number when compared to p-CTR treated diabetic animals: capillaries/HPF: p-SN treated ischemic side: 253±9; p-CTR treated ischemic side: 212±7; p<0.005; n = 10.

We also observed an increase of α-SMA-positive vessels in animals treated by SN gene therapy, consistent with induction of arteriogenesis (figure 4B): α-SMA-positive vessels/HPF: p-SN treated ischemic side: 2.7±0.1; p-CTR treated ischemic side: 2.0±0.2; p<0.01; n = 10.

### SN stimulates endothelial cell migration in an aortic ring assay

**Figure 5:**
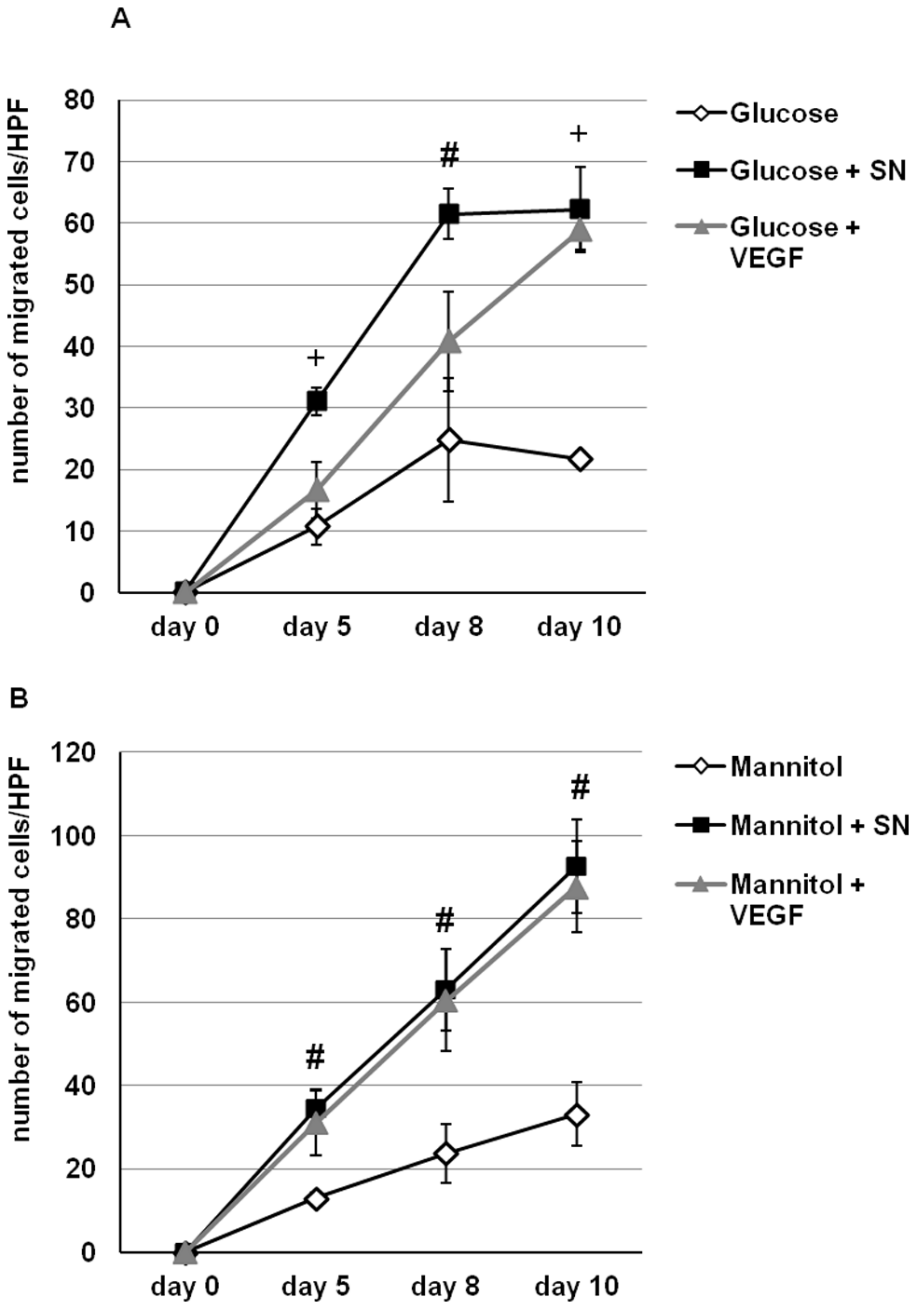
SN induces outgrowth of ECs from aortic rings. Incubation of aortic rings with SN resulted in a significant outgrowth of EC from explanted aortic rings under hyperglycemic conditions (A) and with mannitol as osmotic control (B). The observed effects in our study were comparable or even superior to VEGF used as positive control. +, p<0.01 and #, p<0.05 vs. CTR.

In addition to *in vivo* studies we performed a mouse aortic ring assay as an *ex-vivo* method to study the influence of SN on EC migration under hyperglycemic conditions. After 10 days of incubation the SN treated group showed a significant increase in the outgrowth of endothelial cells from aortic rings compared to controls (number of migrated ECs day 10: glucose: 21.7±1.3 vs. glucose + SN 10 ng/ml: 62.2±3.1, p<0.01 vs. glucose; glucose + VEGF 50 ng/ml: 58.9±7, p<0.01 vs. glucose; n = 3, figure 5A). Mannitol served as osmotic negative control (number of migrated ECs day 10: mannitol: 33.2±7.7 vs. mannitol + SN 10 ng/ml: 92.7±10.9, p<0.05 vs. mannitol; mannitol + VEGF 50 ng/ml: p<0.05 vs. Mannitol; n = 3, figure 5B). After 5 and 8 days under glucose treatment, only SN initiated a significant outgrowth of ECs from aortic rings, whereas VEGF under this conditions did not (number of migrated EC day 5: glucose: 10.7±2.9 vs. glucose + SN 10 ng/ml: 31±4.5, p<0.01 vs. glucose; glucose + VEGF 50 ng/ml: 16.7±2.2; day 8: glucose: 24.9±10.1 vs. glucose + SN 10 ng/ml: 61.5±8, p<0.05 vs. glucose; glucose + VEGF 50 ng/ml: 40.8±4.1; n = 3, figure 5A). These data indicate that SN, comparable to, or (in early time points) even stronger than VEGF stimulates EC outgrowth in the *ex-vivo* aortic ring assay under hyperglycemic conditions.

### SN exerts proliferative and anti-apoptotic effects on HUVECs under hyperglycemic conditions

**Figure 6:**
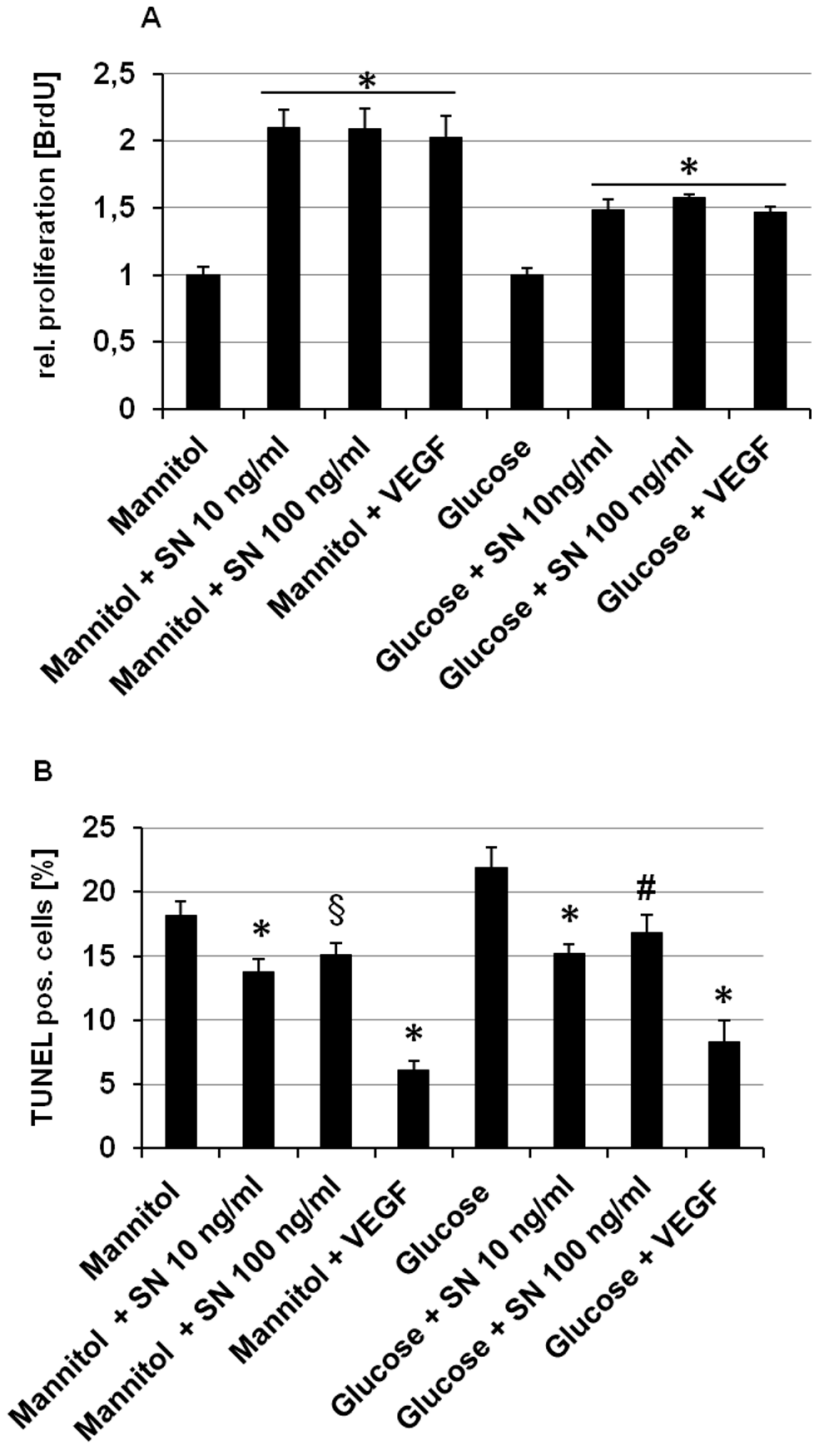
SN stimulates proliferation and reduces apoptosis of HUVECs under hyperglycemic conditions. (A) HUVECs were cultured with medium containing mannitol or glucose and stimulated with SN 10 ng/ml, SN 100 ng/ml and VEGF 50 ng/ml. Proliferation was determined using a BrdU assay. Proliferation was stimulated by SN and VEGF to a comparable extent even under hyperglycemic conditions. *, p<0.001 vs. mannitol or glucose respectively. (B) Apoptotic cells were identified as TUNEL positive cells and are shown as percentage of the total cell number (obtained by DAPI staining). SN and VEGF significantly inhibited apoptotic reactions even under hyperglycemic conditions. *, p<0.001; #, p<0.05; §, p<0.005 vs. mannitol or glucose respectively.

To investigate the role of SN on EC proliferation under hyperglycemia BrdU-uptake was measured. Treatment of HUVEC with SN resulted in a significant increase of proliferation (rel. BrdU uptake glucose vs. glucose + SN 10 ng/ml: 1.49±0.1, p<0.001; n = 3; figure 6A). The observed effects were comparable to VEGF, which served as positive control. Although observed effects were smaller than EC proliferation with SN or VEGF using the osmotic control mannitol (rel. BrdU uptake mannitol vs. mannitol + SN 10 ng/ml: 2.1±0.1, p<0.001; n = 3), SN was capable of inducing significant EC proliferation under hyperglycemic conditions.

For investigating the effect of SN on HUVEC apoptosis under hyperglycemic conditions, cells were starved and stained with TUNEL and DAPI. SN significantly inhibited HUVEC apoptosis with a maximum effect at 10 ng/ml (% TUNEL positive cells of DAPI positive cells: glucose 21.9±1.6 vs. glucose + SN 10 ng/ml 15.2±0.7, p<0.001; n = 3; figure 6B). VEGF served as positive control (% TUNEL positive cells of DAPI positive cells: glucose + VEGF 8.3±1.7, p<0.001 vs. glucose only; n = 3). Similar results for anti-apoptotic effects were observed for SN and VEGF when cells were cultured with mannitol.

### SN induces HUVEC migration and MAPK, AKT, eNOS and EGF receptor activation under hyperglycemic conditions

**Figure 7:**
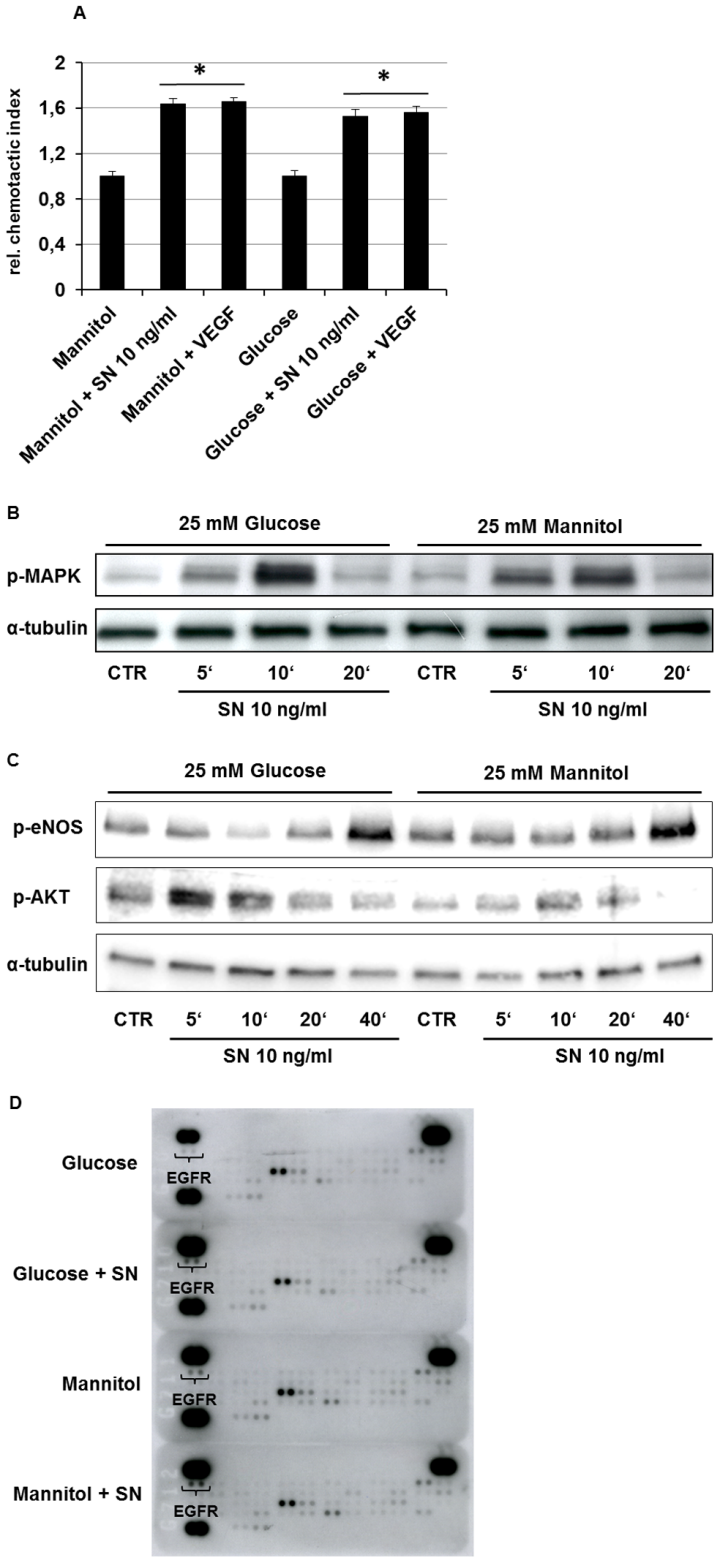
SN induces HUVEC migration and induces MAPK, AKT, eNOS and EGF receptor activation under hyperglycemic conditions. (A) HUVECs were cultured with medium containing mannitol or glucose and stimulated with SN 10 ng/ml and VEGF 50 ng/ml. Migration was determined as movement of cells within a membrane towards attracting substances and is given as relative (to control) chemotactic index. *, p<0.001 vs. mannitol or glucose respectively. (B) As shown in this representative western blot hyperglycemia didn't affect activation of the MAPK-ERK1/2 signaling pathway by SN. (C) HUVECs stimulation with SN 10 ng/ml for 40 minutes activated eNOS under high glucose as well as under mannitol control. AKT activation mediated by SN is shown after 5 and 10 minutes under diabetic and control conditions. Under high glucose, AKT stimulation by SN is even stronger compared to mannitol control. (D) SN 10 ng/ml stimulation for 20 minutes revealed phosphorylation of EGF receptor (EGFR) under high glucose as well as under mannitol osmotic control conditions.

Since angiogenesis is an orchestrated process requiring migration of endothelial cells before proliferation we performed chemotaxis assays using a modified Boyden chamber. Our data show that even under diabetic conditions SN induces migration of HUVEC similar to VEGF (rel. chemotaxis index glucose vs. glucose + SN 10 ng/ml: 1.52±0.061, p<0.001; n = 3; figure 7A). Similar results were obtained for cells cultured with mannitol.

To determine if hyperglycemia might impair activation of intracellular signal transduction pathways by SN we analyzed activation of the MAPK-Erk1/2 signaling axis by western blot assays and observed that SN-induced ERK activation was not influenced by experimental hyperglycemia (figure 7B). As the PI3K/AKT and NO signaling pathways play crucial roles in angiogenesis, western blot analysis were performed and revealed phosphorylation of eNOS after 40 minutes and AKT after 5 and 10 minutes of stimulation with SN 10 ng/ml. Under high glucose conditions, AKT activation with SN was even stronger compared to mannitol control (figure 7C). For further investigation of receptors involved in SN signaling under diabetic conditions, a RTK array was performed and revealed phosphorylation of epidermal growth factor receptor (EGFR) by stimulation with SN 10 ng/ml for 20 minutes (figure 7D).

## Discussion

Recently we showed that SN gene therapy induces therapeutic angiogenesis in rodent hind limb and myocardial ischemia models [18], [19]. In the present investigation we could extend those observations showing the effectiveness of SN gene therapy in a setting of high vascular risk, i.e. under chronic diabetic conditions. SN gene therapy showed robust improvement of limb perfusion and reduced limb tissue necrosis. Increase of capillaries and arteries by SN indicates induction of angiogenesis and arteriogenesis by SN also in this in-vivo model. Additionally, SN exerted positive effects on ECs in-vitro even under hyperglycemic conditions.

Despite a large number of positive preclinical and early clinical studies, the results of growth-factor based pro-angiogenic clinical trials for PAD finally were negative in a large randomized phase III study using FGF-1 gene therapy [8]–[12], [22], [23]. Why did this TAMARIS study fail? The most important difference between animal and human studies probably lies in the fact that most of the preclinical studies were conducted in healthy animals with normal angiogenic response, whereas patients in clinical trials with no options for revascularization usually are characterized by chronic ischemia and mostly several vascular risk factors like diabetes, hypercholesterolemia or hypertension. Especially diabetes seems to impair the angiogenic response dramatically as shown in previous studies [7] and also in this work where we could demonstrate that animals only recover to a LDPI perfusion ratio ischemic versus control leg 4 weeks after ischemia of approximately 0.45 whereas non-diabetic mice usually recover to a ratio between 0.7 and 0.8. Prior reports about the effect of supplementation of angiogenic factors in diabetic animal models of limb ischemia to augment the development of new vessels have been conflicting. Whereas VEGF significantly improved the blood flow in ischemic hind limbs in normal animals [24], it failed to enhance the blood flow in ischemic limbs of diabetic animals [25]. Conversely, other reports demonstrated that angiogenic therapy with VEGF results in an amelioration of blood flow in diabetic mice [7].

Based on our prior works [15]–[19] and on our actual *in vitro* data, where we found that SN exerts strong positive effects on ECs exposed to high glucose concentrations, we reasoned that SN gene therapy might improve blood flow to an ischemic hind limb in the setting of diabetes. We therefore used a well established model of hind limb ischemia in mice injected with STZ which develop a form of diabetes with features similar to those of the human diabetes mellitus type-I. STZ injections induced hyperglycemia, as evidenced by blood glucose measurement. Although clinical trials using plasmids have produced mixed results, we used local gene therapy instead of protein therapy, because of the longer expression period and due to the fact that localized therapy provides the possibility to induce regional angiogenesis processes at the site of injection (i.e. enhance vessel densities in ischemic legs) while not disturbing distant organs (i.e. inducing retinopathy by stimulation of angiogenesis in eyes).

Interestingly, the treatment of diabetic mice with local injection of p-SN showed a significant and huge enhancement in functional neovascularization, as indicated by accelerated recovery of tissue perfusion measured by LDPI and indicated by reduction of necrosis in ischemic legs of diabetic mice. 40% of mice treated with the control plasmid experienced autoamputation, which was reduced by SN gene therapy to 20%. As mice with autoamputation were excluded from LDPI studies restoration of blood flow rates between the SN-treated and the control group may be even underestimated. The improvement in blood flow and clinical outcome may be attributed to enhanced collateralization of blood vessels as we found increased numbers of capillaries and arterioles/arteries significantly after SN therapy, which complement our findings on clinical outcome and blood flow measurements. As diabetes promotes vascular rarefaction and diminishes collateral vessel development in peripheral tissues [26], SN gene therapy might be a reasonable therapeutic option.

*In vitro* SN induced robust effects on ECs despite hyperglycemic conditions: we found that SN is capable of promoting proliferation and migration of HUVECs comparable to the prototypical angiogenic cytokine VEGF and stimulates MAPK (ERK), AKT and eNOS activation with AKT activation even stronger than in non-hyperglycemic cells. Given the anti-apoptotic effects of AKT in ECs it is conceivable that SN-induced AKT activation might contribute to inhibition of hyperglycemia-induced apoptosis in ECs by SN. In a previous work [19] we found that SN stimulates receptors for potent angiogenic cytokines (VEGF, fibroblast growth factor, FGF, and insulin-like growth factor, IGF) in coronary ECs. In this work we observed in HUVECs that epidermal growth factor receptor (EGFR) was stimulated by SN in a profiler assay under high glucose conditions as well as in the mannitol control. Interestingly, EGFR was reported recently to be responsible for improvement of cardiac recovery in diabetic hearts, as diabetes led to attenuated dimerization and phosphorylation of the EGFR [27]. Investigations of mRNA levels showed no influence of SN on regulation of growth factors/receptors activated by SN (figure S1A-F in File S1). As thioredoxin interacting protein (TXNIP) was shown to contribute to impaired angiogenesis in diabetes [28], [29], we investigated whether SN might inhibit TXNIP on mRNA level. We however could not find regulation of TXNIP by SN alone or in combination with VEGF (figures S2A and S2C in File S1). Another possible mechanistic way of SN action was excluded by investigation of proto-oncogene serine/threonine-protein kinase (PIM1), which has been reported to be crucial for in vitro angiogenesis [30] and in vivo cell survival under diabetic conditions (figure S2B in File S1) [31], [32]. We therefore suggest that SN-induced AKT activation and EGFR activation under hyperglycemic conditions might contribute to reduced glucose-induced apoptosis and stimulating effects on endothelial cell sprouting in an aortic ring assay ex-vivo under hyperglycemic conditions. These in-vitro and ex-vivo effects of SN might explain the robust benefits of SN gene therapy in our in-vivo model.

Nevertheless, this study has some limitations including lack of use of different doses of SN plasmid and use of animal models mimicking type-2 DM, which should be addressed in future studies.

## Conclusions

Our study demonstrates that the angiogenic factor SN exerts beneficial effects like stimulation of proliferation and migration as well as inhibition of apoptosis in ECs cultured under hyperglycemic conditions. Notably SN strongly stimulated AKT activation and EGFR activation under hyperglycemia. In-vivo SN induced robust effects in a model of hind limb ischemia in type 1 diabetic mice like inhibition of necrosis and amputation as well as improvement of blood flow recovery by induction of angiogenesis and arteriogenesis. Our data suggested that SN therapy might be used as a promising stand-alone or adjunctive approach for therapeutic neovascularization in diabetic limb ischemia.

## Supporting Information

File S1 **Supporting text and figures.** **Figure S1. SN effect on mRNA expression of growth factors/receptors under high glucose.** None of the analyzed growth factors or their corresponding receptors were significantly regulated on mRNA level by stimulation with SN under high glucose conditions. The following growth factors and growth factor receptors have been investigated: VEGF (A), VEGF receptor 2 (VEGFR2) (B), basic fibroblast growth factor (bFGF) (C), FGF receptor 3 (FGFR3) (D), insulin-like growth factor receptor (IGFR) (E) and epidermal growth factor receptor (EGFR) (F). **Figure S2. SN effect on mRNA expression of TXNIP and PIM1 under high glucose.** (A) shows thioredoxin-interacting protein (TXNIP) mRNA levels of HUVECS under high glucose and stimulation with SN 10 ng/ml for 12 hours. SN showed no effect on TXNIP or Proto-oncogene serine/threonine-protein kinase (PIM1) mRNA expression (B). SN showed no effect on TIXNIP expression under stimulation by SN in combination with VEGF (C).
(DOCX)
