# Supplementary material for: Secretoneurin Gene Therapy Improves Blood Flow in an Ischemia Model in Type 1 Diabetic Mice by Enhancing Therapeutic Neovascularization
Source: PLoS One. 2013 Sep 23;8(9):e74029. doi: 10.1371/journal.pone.0074029 (PMC3781158; doi:10.1371/journal.pone.0074029)
Supplement: File S1 — Supporting text and figures. Figure S1. SN effect on mRNA expression of growth factors/receptors under high glucose. None of the analyzed growth factors or their corresponding receptors were significantly regulated on mRNA level by stimulation with SN under high glucose conditions. The following growth factors and growth factor receptors have been investigated: VEGF (A), VEGF receptor 2 (VEGFR2) (B), basic fibroblast growth factor (bFGF) (C), FGF receptor 3 (FGFR3) (D), insulin-like growth factor receptor (IGFR) (E) and epidermal growth factor receptor (EGFR) (F). Figure S2. SN effect on mRNA expression of TXNIP and PIM1 under high glucose. (A) shows thioredoxin-interacting protein (TXNIP) mRNA levels of HUVECS under high glucose and stimulation with SN 10 ng/ml for 12 hours. SN showed no effect on TXNIP or Proto-oncogene serine/threonine-protein kinase (PIM1) mRNA expression (B). SN showed no effect on TIXNIP expression under stimulation by SN in combination with VEGF (C). (DOCX) [file pone.0074029.s001.docx]

**Supplemental Data**

**Methods**

**Real-time qPCR**

HUVECs (Promocell) between passage 2 and 5 were used and cultured in EBM-2 medium (Lonza). 24 hours prior to experiments cells were incubated with 25 mM D-Glucose or 25 mM mannitol as osmotic negative control. Cells were incubated with SN (10 or 100 ng/ml) and/or VEGF (10 or 100 ng/ml) for different time intervals. After washing, RNA was isolated with RNeasy Mini Kits (Quiagen) as suggested by the manufacturer. 1 μg of total RNA was transcribed using Superscript TM First-Strand Synthesis System (Invitrogen). cDNA was finally used as template for real time PCR using a BioRad C 1000 cycler with CFX96 optical reaction module and SsoFastTM Eva Green® Supermix (BioRad). For amplification, the following primer pairs were used:

Human VEGF-A: fwd GTG CCC ACT GAG GAG TCC A; rev TCC TAT GTG CTG GCC TTG GT; Human VEGFR2: fwd TCC TGT ATG GAG GAG GAG GA; rev TAC CAC TGT CCG TCT GGT TG; Human bFGF: fwd AGC GAC CCT CAC ATC AAG CTA; rev CCA GGT AAC GGT TAG CAC ACA CT; Human FGFR3: fwd AGC TCA CCT TCA AGG ACC TG
rev GCG ATC TTC ATC ACG TTG TC; Human IGF: fwd TGG ATG CTC TTC AGT TCG TG
rev GGT GCG CAA TAC ATC TCC AG; Human IGFR: fwd TCC AAG GAT GCA CCA TCT TC; rev AAT GGC GGA TCT TCA CGT AG; Human TXNIP: fwd ATC CCT GAT ACC CCA GAA GC; rev TTC TTC CAC ATG CTC ACT GC; Human PIM1: fwd TTC CTT TCG AGC ATG ACG; rev TGG ATT TCT TCG AAG GTT GG; Human EGF: fwd CCA AGT AAC ACC ATT GGA CAT C; rev ATA CAT GCT GCA TCC CAC AG; Human EGFR: fwd GGC CAA ATA CAG CTT TGG TG; rev GGA GGT GCA GTT TTT GAA GTG;

The reaction sequence included 35 sec at 95°C and 10 sec at 60°C for 40 cycles. Relative gene expression was calculated using the Ct method with normalization to HPRT: fwd CCT GGC GTC GTG ATT AGT GAT GA; rev CAA GAC GTT CAG TCC TGT CCA TAA TTA;

**Results**

**Real-time qPCR**

In order to provide a more mechanistic insight of SN action under high glucose conditions in vitro, expressions of mRNA levels in HUVECs were investigated. Supplemental figure 1A to 1E show mRNA levels in HUVECs under high glucose and stimulation with SN 10 ng/ml for 6 and 12 hours. Mannitol has been used as negative control. The following growth factors and growth factor receptors have been investigated: VEGF, VEGF receptor 2 (VEGFR2), basic fibroblast growth factor (bFGF), FGF receptor 3 (FGFR3), insulin-like growth factor receptor (IGFR) and epidermal growth factor receptor (EGFR). IGF and EGF have been analyzed as well, but their expression was below detection level (data not shown). None of the analyzed growth factors nor their corresponding receptors, were significantly regulated on mRNA level by stimulation with SN under high glucose conditions.

Supplemental figure 2A shows thioredoxin-interacting protein (TXNIP) mRNA levels of HUVECs under high glucose and stimulation with SN 10 ng/ml for 12 hours. As reported previously, TXNIP mRNA levels were increased under high glucose conditions, compared to mannitol control [1,2]. Nevertheless, SN showed no further effect on TXNIP mRNA expression. Supplemental figure 2B shows mRNA expression of Proto-oncogene serine/threonine-protein kinase (PIM1) under high glucose and stimulation with SN 10 ng/ml for 12 hours. PIM1 has been reported to be crucial for in vitro angiogenesis [3] and in vivo cell survival under diabetic conditions [4,5]. However, SN stimulation had no significant effect on PIM1 mRNA regulation.

For investigating a possible synergistic effect on TIXNIP expression of SN in combination with VEGF, HUVECS were stimulated under high glucose with SN 10 or 100 ng/ml with or without VEGF 10 and 100 ng/ml for 24 hours. Regulation of TIXNIP mRNA showed no influence from SN or VEGF (supplemental figure 2C).


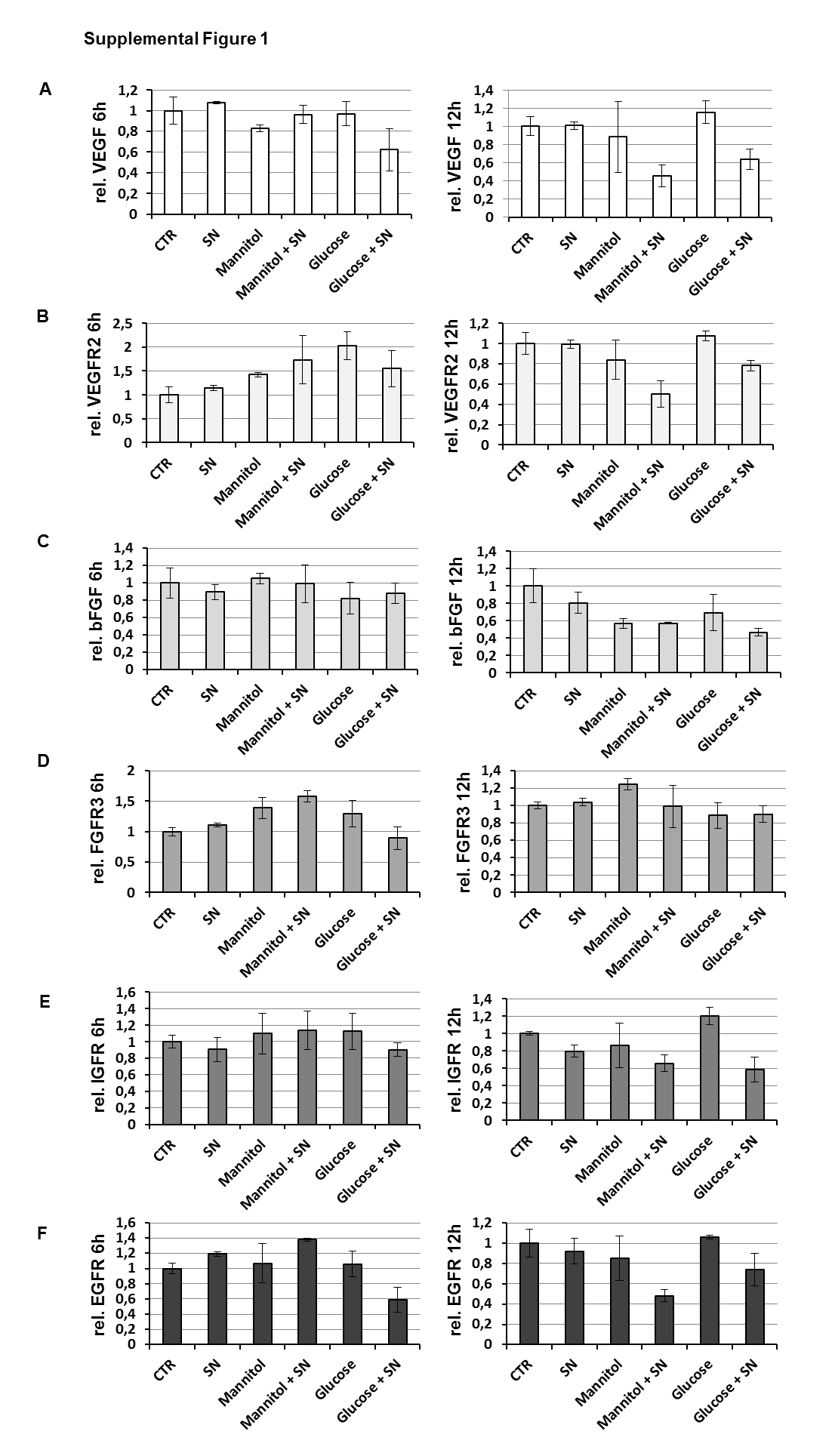


**
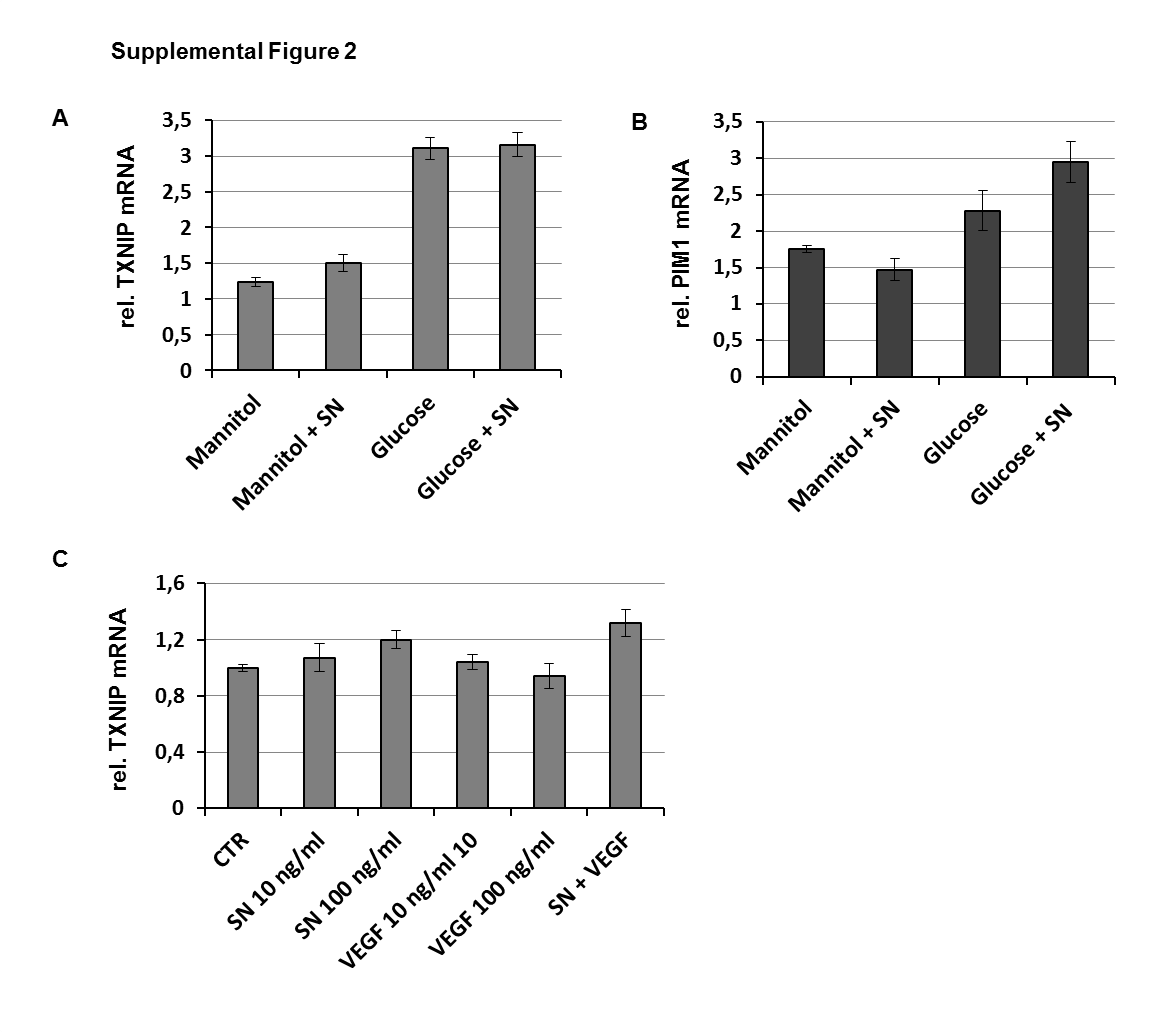
**

**Supplemental figure legends**

**Supplemental figure 1 SN effect on mRNA expression of growth factors/receptors under high glucose**

None of the analyzed growth factors or their corresponding receptors were significantly regulated on mRNA level by stimulation with SN under high glucose conditions. The following growth factors and growth factor receptors have been investigated: VEGF (A), VEGF receptor 2 (VEGFR2) (B), basic fibroblast growth factor (bFGF) (C), FGF receptor 3 (FGFR3) (D), insulin-like growth factor receptor (IGFR) (E) and epidermal growth factor receptor (EGFR) (F).

**Supplemental figure 2 SN effect on mRNA expression of TXNIP and PIM1 under high glucose**

(A) shows thioredoxin-interacting protein (TXNIP) mRNA levels of HUVECS under high glucose and stimulation with SN 10 ng/ml for 12 hours. SN showed no effect on TXNIP or Proto-oncogene serine/threonine-protein kinase (PIM1) mRNA expression (B). SN showed no effect on TIXNIP expression under stimulation by SN in combination with VEGF (C).

**References**

1. Buckle A, Dunn LL, Ng MKC (2007) Hyperglycaemia Inhibits Thioredoxin- Mediated Angiogenesis: Implications for Impairment of Neovascularisation in Diabetes Mellitus. Heart, Lung and Circulation 16: S214–S215.

2. Li X, Rong Y, Zhang M, Wang XL, LeMaire SA, Coselli JS, Zhang Y, Shen YH (2009) Up- regulation of thioredoxin interacting protein (Txnip) by p38 MAPK and FOXO1 contributes to the impaired thioredoxin activity and increased ROS in glucose-treated endothelial cells. Biochem Biophys Res Commun 381:660–665.

3. Zippo A, De Robertis A, Bardelli M, Galvagni F, Oliviero S (2004) Identification of Flk-1 target genes in vasculogenesis: Pim-1 is required for endothelial and mural cell differentiation in vitro. Blood 103(12):4536-44.

4. Katare R, Caporali A, Zentilin L, Avolio E, Sala-Newby G, Oikawa A, Cesselli D, Beltrami AP, Giacca M, Emanueli C, Madeddu P (2011) Intravenous gene therapy with PIM-1 via a cardiotropic viral vector halts the progression of diabetic cardiomyopathy through promotion of prosurvival signaling. Circ Res 108(10):1238-51.

5. Katare R, Oikawa A, Cesselli D, Beltrami AP, Avolio E, Muthukrishnan D, Munasinghe PE, Angelini G, Emanueli C, Madeddu P (2013) Boosting the pentose phosphate pathway restores cardiac progenitor cell availability in diabetes. Cardiovasc Res 97(1):55-65.
